# Fungal and Bacterial Loads: Noninvasive Inflammatory Bowel Disease Biomarkers for the Clinical Setting

**DOI:** 10.1128/mSystems.01277-20

**Published:** 2021-03-23

**Authors:** G. Sarrabayrouse, A. Elias, F. Yáñez, L. Mayorga, E. Varela, C. Bartoli, F. Casellas, N. Borruel, C. Herrera de Guise, K. Machiels, S. Vermeire, C. Manichanh

**Affiliations:** a Department of Gastroenterology, Vall d’Hebron Research Institute, Barcelona, Spain; b CIBERehd, Instituto de Salud Carlos III, Madrid, Spain; c Translational Research Center for Gastrointestinal Disorders (TARGID), Department of Chronic Diseases Metabolism and Ageing, KU Leuven, Leuven, Belgium; d Department of Gastroenterology and Hepatology, University Hospitals Leuven, KU Leuven, Leuven, Belgium; Vanderbilt University Medical Center

**Keywords:** Crohn’s disease and ulcerative colitis, microbial load, machine learning algorithm, diagnosis and prognosis, inflammatory bowel disease, prediction

## Abstract

Microbiome sequence data have been used to characterize Crohn's disease (CD) and ulcerative colitis (UC). Based on these data, we have previously identified microbiomarkers at the genus level to predict CD and CD relapse. However, microbial load was underexplored as a potential biomarker in inflammatory bowel disease (IBD). Here, we sought to study the use of fungal and bacterial loads as biomarkers to detect both CD and UC and CD and UC relapse. We analyzed the fecal fungal and bacterial loads of 294 stool samples obtained from 206 participants using real-time PCR amplification of the ITS2 region and the 16S rRNA gene, respectively. We combined the microbial data with demographic and standard laboratory data to diagnose ileal or ileocolonic CD and UC and predict disease relapse using the random forest algorithm. Fungal and bacterial loads were significantly different between healthy relatives of IBD patients and nonrelated healthy controls, between CD and UC patients in endoscopic remission, and between UC patients in relapse and non-UC individuals. Microbial load data combined with demographic and standard laboratory data improved the performance of the random forest models by 18%, reaching an average area under the receiver operating characteristic curve (AUC) of 0.842 (95% confidence interval [CI], 0.65 to 0.98), for IBD diagnosis and enhanced CD and UC discrimination and CD and UC relapse prediction. Our findings show that fecal fungal and bacterial loads could provide physicians with a noninvasive tool to discriminate disease subtypes or to predict disease flare in the clinical setting.

**IMPORTANCE** Next-generation sequence data analysis has allowed a better understanding of the pathophysiology of IBD, relating microbiome composition and functions to the disease. Microbiome composition profiling may provide efficient diagnosis and prognosis tools in IBD. However, the bacterial and fungal loads of the fecal microbiota are underexplored as potential biomarkers of IBD. Ulcerative colitis (UC) patients have higher fecal fungal and bacterial loads than patients with ileal or ileocolonic CD. CD patients who relapsed harbor more-unstable fungal and bacterial loads than those of relapsed UC patients. Fecal fungal and bacterial load data improved prediction performance by 18% for IBD diagnosis based solely on clinical data and enhanced CD and UC discrimination and prediction of CD and UC relapse. Combined with existing laboratory biomarkers such as fecal calprotectin and C-reactive protein (CRP), microbial loads may improve the diagnostic accuracy of IBD and of ileal CD and UC disease activity and prediction of UC and ileal CD clinical relapse.

## INTRODUCTION

Fungal microbiota, also known as mycobiota, refers to the community of fungi distributed on and within the human body (1). Sequence data analysis (2) estimates that the mycobiota accounts for approximately 0.03% to 0.1% of the gut microbiota and also reveals that it is composed mainly of *Saccharomyces*, *Malassezia*, and *Candida* (3, 4).

In addition to dysbiosis of the bacterial community composition, patients with inflammatory bowel disease (IBD) (3, 5) present an unbalanced fungal community (6–8), which suggests that fungi also play a role in IBD pathogenesis. A decrease in biodiversity and the proportion of Saccharomyces cerevisiae (S. cerevisiae) and an increased proportion of Candida albicans (C. albicans) are associated with IBD, without a clear distinction between Crohn’s disease (CD) and ulcerative colitis (UC), the two main forms of IBD. Furthermore, an interaction between fungi and bacteria has emerged as a potential key player in the pathophysiology of IBD (9), however, without a demonstration of a causative role.

Many studies addressing the relationship between IBD and the microbiome have focused on the analysis of bacterial composition using 16S rRNA sequencing (5, 8) and on the composition of fungi using either 18S rRNA or internal transcribed spacer (ITS1 or ITS2) sequencing. These studies provided important insights into the taxonomic profiling of microbiota but did not shed light on its biomass. We hypothesize that the influence of microbiota on host physiology in IBD also relies on the microbial load and not merely on the type and relative abundance of microorganisms interacting with the host.

Here, we performed a retrospective study using fecal fungal and bacterial loads to characterize CD and UC patients during disease progression, and we compared these data with those from two groups of healthy controls (first-degree relatives of the IBD patients and nonrelated healthy controls [NRHC]). Using random forest—a supervised learning algorithm—we also combined demographic and laboratory variables with fungal and bacterial loads to evaluate their capacity to predict IBD, IBD subtypes, and disease flare.

## RESULTS

### Fungal and bacterial loads.

**FIG 1:**
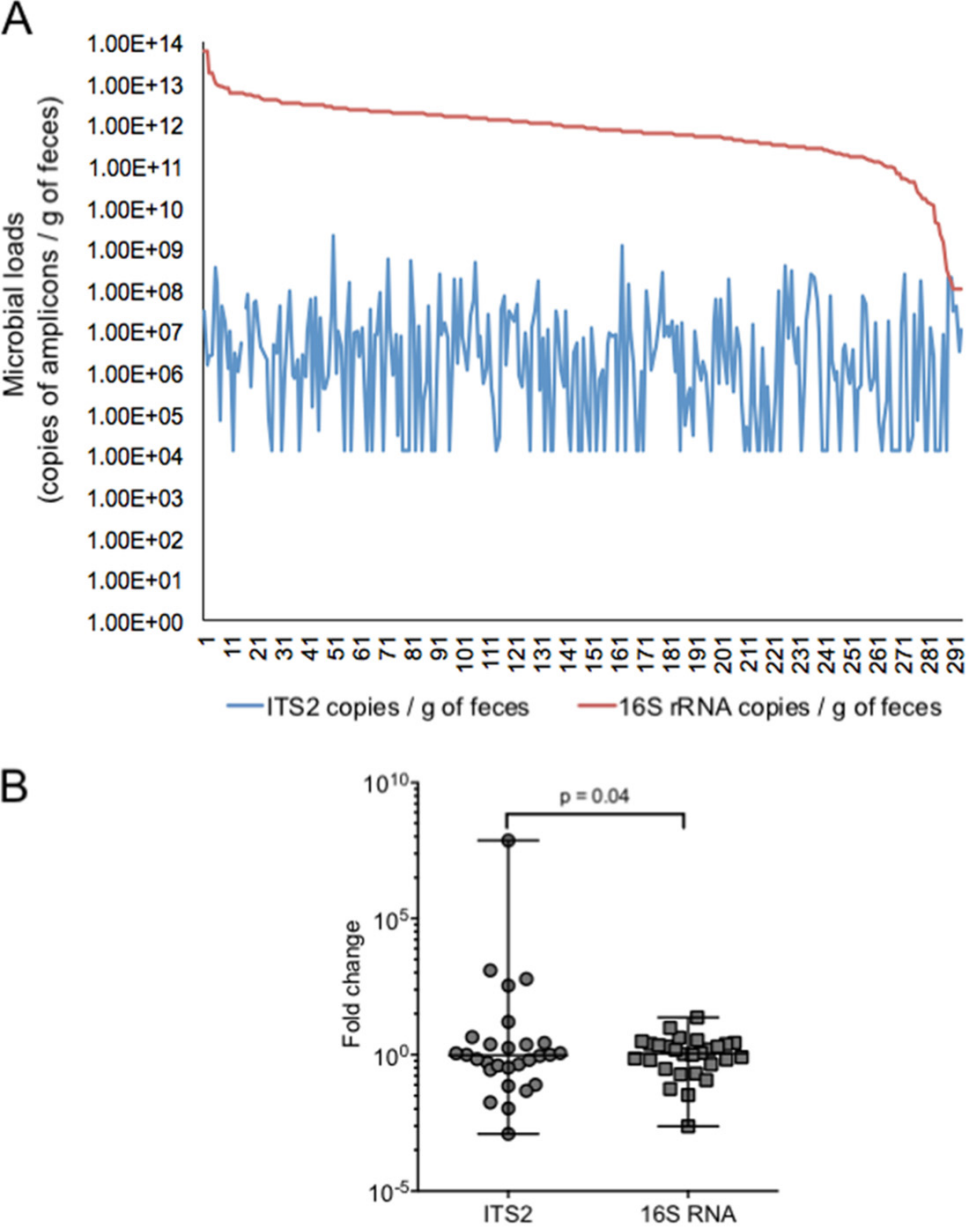
Variation of fungal and bacterial loads. Quantification was performed using real-time PCR amplification of the ITS2 region and the 16S rRNA gene on stool samples. (A) Variation over the population of study. Values along the *x* axis represent the number of samples analyzed. (B) Variation over time (1 month apart). Levene’s test was applied to check the homogeneity of variance between groups.

To assess the fungal and bacterial loads (proxy for the number of fungal and bacterial cells) of the 294 stool samples collected from healthy controls and IBD patients, we quantified the ITS2 region (proxy for fungal load) and the 16S rRNA gene (proxy for bacterial load) by quantification by real-time PCR (qPCR). The number of copies per gram of stool varied from 1.25E+4 to 2.06E+9 for the ITS2 region and from 1E+8 to 5.7E+13 for the 16S rRNA gene, with an average copy number of 3.84E+07 and 1.9E+12 per gram of stool, respectively, leading to an average 16S-to-ITS2 ratio of 4.95E+04 (Fig. 1A). These results suggest that fungal load was far lower than bacterial load, with an estimated 1 fungal cell per 2,000 bacterial cells. However, an exact calculation of fold difference between bacterial and fungal cells was not possible because an accurate estimate of the number of copies of the 16S rRNA gene per bacterial cell (estimated to range from 1 to 15 copies) (10) and of the ITS2 region per fungal cell (estimated to range from 5 to 200 copies) (11, 12) is, so far, not possible in stool samples, due in part to the complexity of each microbial community.

To compare the temporal evolution of fungal and bacterial loads, we examined the copy number of ITS2 and 16S rRNA in stool samples of nonrelated healthy controls (*n* = 28) collected at two time points (initial and 1 month later [M1]). The fold change of the fungal or bacterial load between the two time points showed a greater variation of former community (Levene’s test, *P* = 0.04; removing possible outliers, we still kept significant differences, *P* = 0.042) (Fig. 1B). Interestingly, using Spearman’s correlation test, we did not find a significant correlation (*P* = 0.07; *r* = 0.34 for baseline; *P* = 0.215; *r* = 0.242 for M1) between the copy numbers of ITS2 and 16S rRNA, thereby suggesting that the two microbial communities may vary independently in number of cells in order to maintain gastrointestinal (GI) tract homeostasis. Altogether, our findings confirmed previous studies showing that the fungal compartment of the gut microbiota is less abundant and less stable than the bacterial counterpart (4) and thus validated our approach using qPCR of ITS2 and 16S rRNA for the analysis of the fungal and bacterial loads in stool.

### Higher fungal and lower bacterial load in UC relatives.

**FIG 2:**
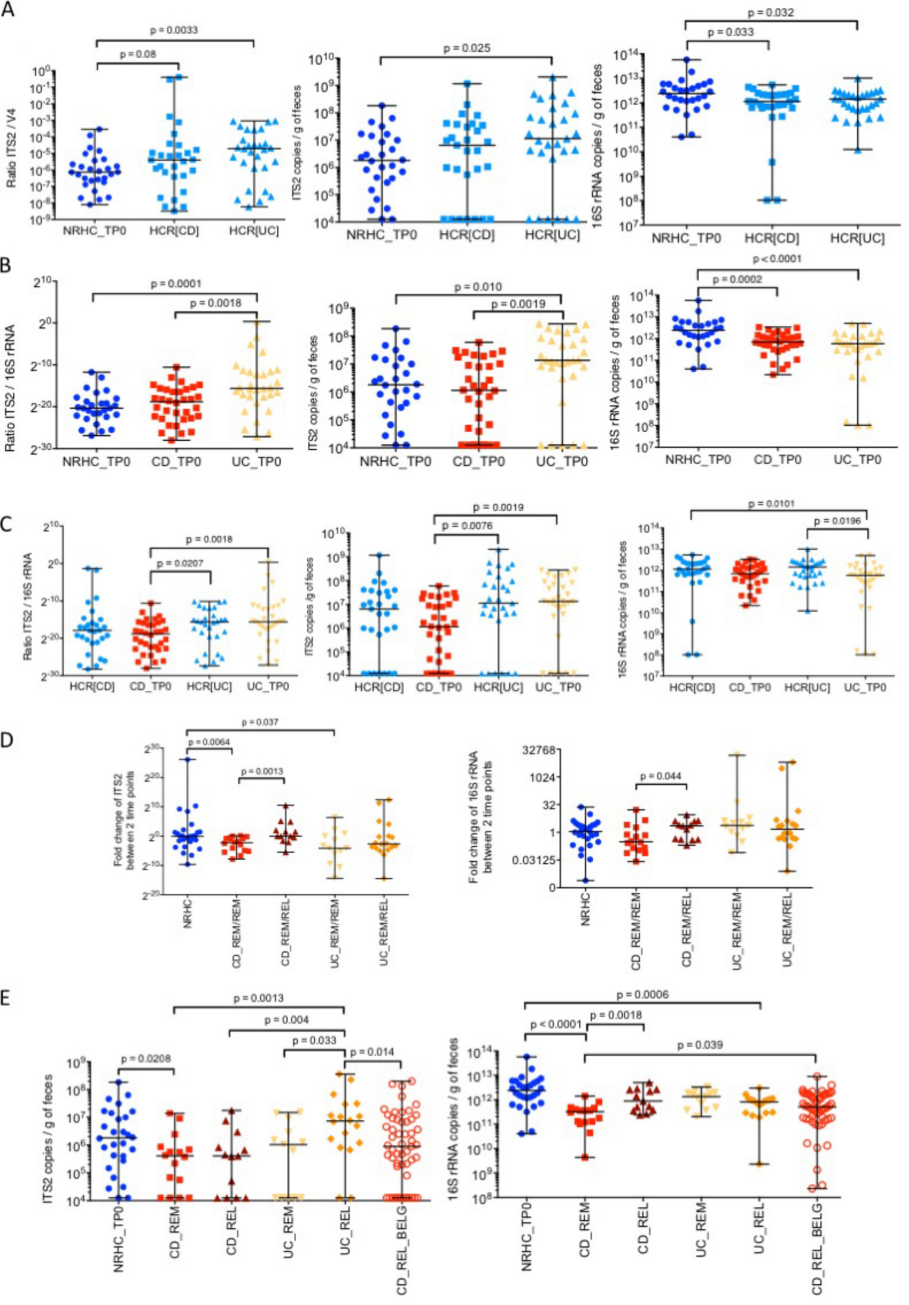
Comparison of fungal and bacterial loads in healthy subjects and IBD patients. Quantification was performed using real-time PCR amplification of the ITS2 region and the 16S rRNA gene on stool samples. (A) Healthy controls related to CD (HCR[CD], *n* = 29) and UC patients (HCR[UC], *n* = 29) with higher fungal and bacterial loads compared to nonrelated controls (NRHC, *n* = 28). (B) UC patients harbored higher fungal and bacterial loads than NRHC (*n* = 28) and CD patients (*n* = 34). (C) Healthy controls related to IBD patients (*n* = 65) presented similar fungal loads as their sick relatives (*n* = 56), and only UC patients (*n* = 31) showed a lower bacterial load than their unaffected relatives (*n* = 29). (D) CD patients (*n* = 34) presented a more unstable fungal community and an increase in fungal and bacterial loads when they experienced relapse (*n* = 21). (E) Validation of the differences in fungal load between UC (*n* = 31) and CD using a Belgian CD cohort (*n* = 55). Statistical analyses were performed using the Mann-Whitney test. Abbreviations are as in the Fig. 3 legend.

Previous studies have reported that first-degree relatives of IBD patients present a dysbiotic gut microbial community composition and lower load of Faecalibacterium prausnitzii compared to nonrelated controls (13, 14). Our analysis of the Spanish cohort indicated that first-degree relatives of UC patients presented a higher fungal/bacterial load ratio compared to nonrelated healthy controls (*n* = 29) (Mann-Whitney test, *P* = 0.003), which was due to a greater presence of fungi and a lower level of bacteria (Fig. 2A). Although not significant, a similar trend was observed between nonrelated healthy controls and unaffected CD relatives (*n* = 29) (Mann-Whitney test, *P* = 0.08). Interestingly, no significant differences in the fungal and bacterial loads were found between the two groups of IBD relatives.

### Higher fungal and lower bacterial load in UC patients in clinical remission.

In the Spanish cohort, UC patients (*n* = 31) in remission had a higher fungal/bacterial load ratio compared to nonrelated healthy controls (*n* = 28) (Mann-Whitney test, *P* = 0.0001) and to CD patients in remission (*n* = 34) (Mann-Whitney test, *P* = 0.0018) (Fig. 2B). In contrast to the nonrelated healthy controls, the relatives of UC patients only showed a significantly larger amount of bacteria compared to their affected family member (Mann-Whitney test, *P* = 0.0196). This difference was not observed between unaffected relatives of CD patients and their affected family members (Fig. 2C). Moreover, UC relatives had a higher fungal/bacterial load ratio than the CD patients (Mann-Whitney test, *P* = 0.02), due mainly to a higher level of fungi in the former (Mann-Whitney test, *P* = 0.0076) (Fig. 2C).

### Fungal and bacterial loads varied with disease course in CD.

Spanish IBD patients were followed up for 1 year. Fecal samples were collected at baseline and then every 3 months. When patients relapsed, they provided their last sample around the time of relapse. The fold change analysis (relapse or remission time point to baseline) of ITS2 and 16S rRNA copies showed that both fungal and bacterial loads increased when CD patients relapsed (median fold change = 2.23) and decreased when patients remained in remission for a year (median fold change = 0.30) (Fig. 2D). In contrast, nonrelated healthy controls presented a median fold change of 0.95 for fungi and 1.1 for bacteria. Moreover, CD patients who relapsed presented a significant difference in fold change for both fungal (Mann-Whitney test, *P* = 0.0013) and bacterial (Mann-Whitney test, *P* = 0.044) loads compared to those who remained in remission (Fig. 2D). A similar temporal instability was not observed in UC patients (Fig. 2D). A decrease in fungal load (median fold change = 0.057) was observed only when UC patients remained in remission. Interestingly, UC patients undergoing a flare presented an increase in the fungal/bacterial load ratio compared to healthy controls (*P* = 0.001), CD patients in remission (*P* = 0.001), CD patients in relapse (*P* = 0.001), and UC patients in remission (*P* = 0.001) (Fig. 2E). This increase is attributed mainly to a burst in fungal load.

We validated some of these findings using another CD cohort from a prior study in Belgium (15) in which patients with active disease underwent ileocecal resection and provided fecal samples for microbial load analysis before surgery (Fig. 2E). We did not find differences in fungal or bacterial loads between the Spanish and Belgian CD patients. However, like the Spanish CD cohort, the Belgian CD patients showed a significantly lower fungal load compared to the Spanish UC patients in relapse (Mann-Whitney test, *P* = 0.014). This observation strengthens the results obtained with the Spanish cohort and points to an absence of geographical differences.

### Fecal calprotectin and CRP as biomarkers of disease activity.

**FIG 3:**
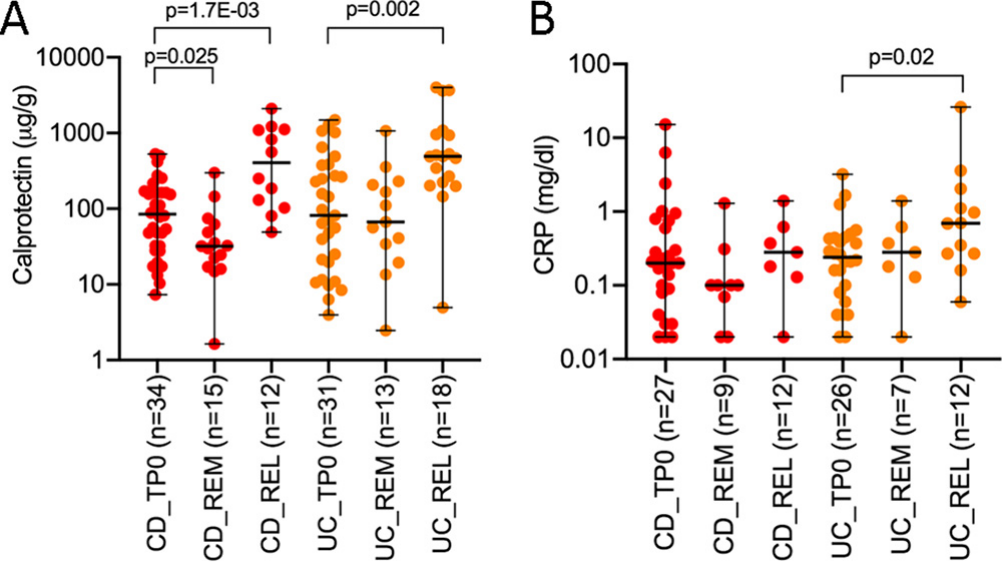
Fecal calprotectin and C-reactive protein (CRP) levels. Fecal calprotectin (A) and CRP (B) were measured on available samples of patients with CD and UC at baseline (TP0) and after 1 year in remission (REM) and at recurrence (REL). The Mann-Whitney test was used to compare differences between groups. CD, Crohn’s disease; UC, ulcerative colitis.

Standard laboratory markers such as fecal calprotectin and C-reactive protein (CRP) were measured on available samples of IBD patients from the Spanish cohort at baseline and then at relapse or after 1 year if the patient remained in remission. Fecal calprotectin was significantly different between remission and relapse for both CD and UC patients, and CRP showed significant differences only between UC at baseline (in remission) and UC at flare (Mann-Whitney, *P* = 0.02) (Fig. 3A and B). As expected, fecal calprotectin and CRP were not significantly different between CD and UC both at remission and at flare.

### No effect of immunosuppressants in fungal or bacterial loads.

Since the utilization of immunosuppressants may impact microbial loads (Table S1), we compared for each group of participants (CD, UC, CD and UC in remission, and CD and UC in relapse) the fungal and bacterial loads between individuals who took immunosuppressants and those who did not. We did not encounter any significant effect of these drugs on the microbial loads using the Wilcoxon test, which may be due to the small cohort size.

10.1128/mSystems.01277-20.2 TABLE S1 Characteristics of participants. Download Table S1, PDF file, 0.08 MB. Copyright © 2021 Sarrabayrouse et al. 2021 Sarrabayrouse et al. https://creativecommons.org/licenses/by/4.0/ This content is distributed under the terms of the Creative Commons Attribution 4.0 International license.

### Predictive value of fungal and bacterial loads.

Efforts channeled into developing a noninvasive test that discriminates disease subtypes or predicts disease flare have been driven by the many disadvantages of endoscopy. Endoscopy, apart from being expensive and uncomfortable for the patients, may not be decisive and therefore may delay treatment decisions. To this end, we used random forest, a supervised learning algorithm, to examine the capacity of fungal and bacterial loads to predict IBD, the two major IBD subtypes (CD and UC), and disease flare. We created several random forest models based on fungal and bacterial load data and, when available, other standard laboratory and demographic data, and we randomly split the population of interest into a training set (2/3) and a test set (1/3).

**FIG 4:**
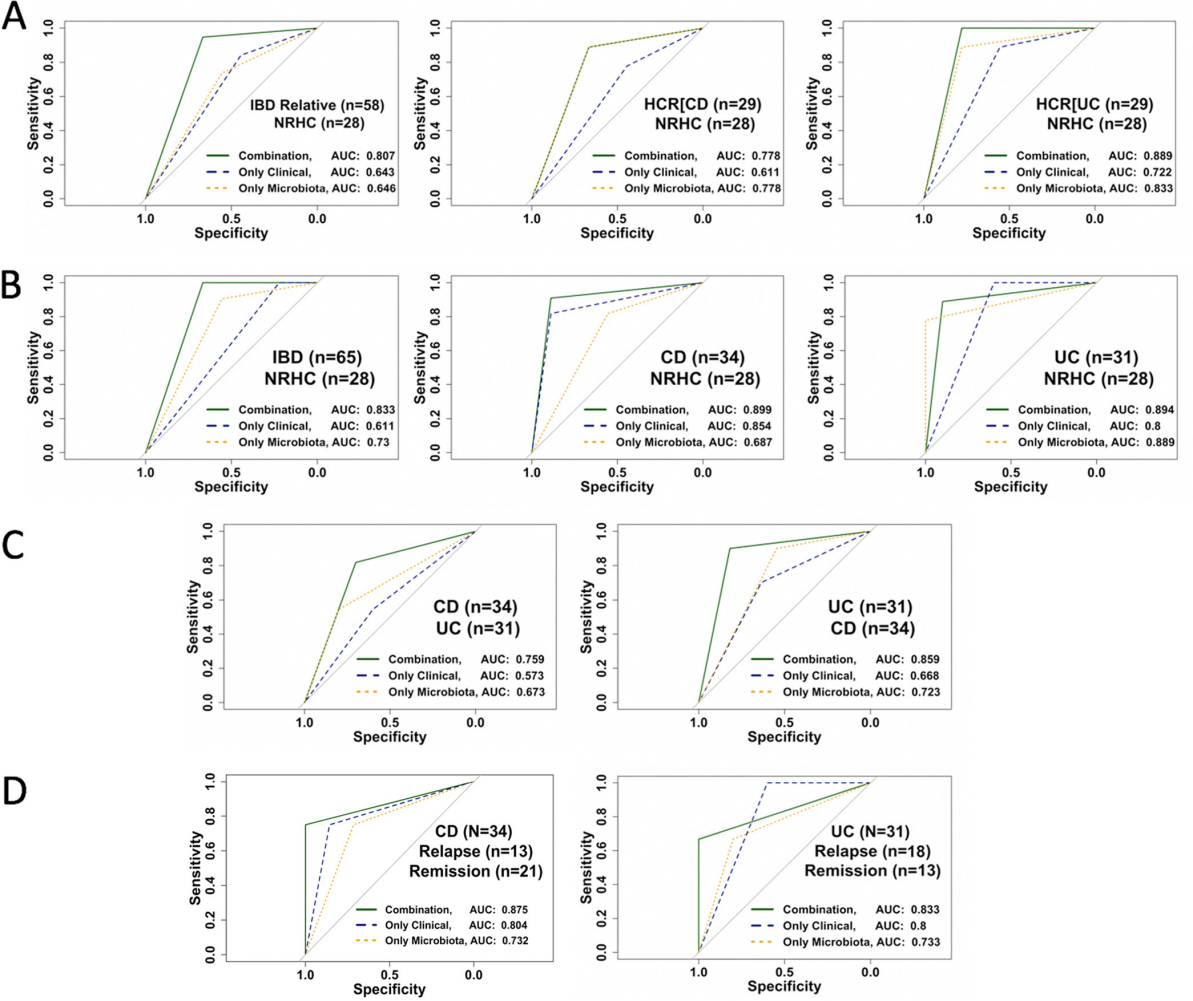
Receiver operating characteristic (ROC) curves for the random forest models. (A) ROC curves for the three control models. (B) ROC curves for IBD versus control model. (C) ROC curves for CD versus UC model. (D) ROC curves for disease relapse model. NRHC, nonrelated healthy controls; HCR[CD], healthy controls related to CD patients; HCR[UC], healthy controls related to UC patients; AUC, area under the curve; Only Clinical, only standard laboratory tests and demographic data; Only Microbiota, 16S rRNA plus ITS2 plus 16S rRNA-to-ITS2 ratio.

To classify the two groups of healthy controls, we first combined the relatives of CD and UC patients (*n* = 58) and used them as the case group. The NRHC group (*n* = 28) was used as the control group. For this classification and due to missing standard laboratory or clinical data as predictor variables for the NRHC subjects, we were limited to microbial data and a few demographic data, such as age, weight, height, body mass index (BMI), gender, and smoking habit. We obtained an area under the receiver operating characteristic (ROC) curve (AUC) of 0.80 (sensitivity of 0.94 and specificity of 0.66; 95% confidence interval [CI], 0.63 to 0.97) (Fig. 4A). Fungal and bacterial load, age, gender, BMI, and smoking habit appeared to be key variables. Interestingly, when we analyzed the two groups of relatives separately from the NRHC subjects, we obtained an AUC of 0.77 (sensitivity of 0.88 and specificity of 0.66; 95% CI, 0.58 to 0.97) with the CD relatives (*n* = 29), and we improved the model with an AUC of 0.89 (sensitivity of 1 and specificity of 0.77; 95% CI, 0.74 to 1) with the UC relatives (*n* = 29) (Fig. 4A). Fungal and bacterial loads, BMI, and smoking habit emerged as important variables for both cases. These results validate our prior findings that the microbial loads of healthy relatives of IBD patients differ from those of nonrelated controls.

To differentiate IBD from healthy controls, we first combined the CD and UC cohorts (*n* = 65) as the case set and used the NRHC cohort (*n* = 28) as the control set. For these analyses, we were again limited to microbial data and a few demographic data such as age, BMI, gender, and smoking habit. The predictive variables that led to the highest AUC of 0.83 with a high sensitivity of 1 and a low specificity of 0.66 (95% CI, 0.67 to 0.99) were fungal and bacterial loads, BMI, and smoking habit (Fig. 4B). However, when we split IBD by subtypes, we obtained an AUC of 0.89 (sensitivity of 0.90 and specificity of 0.88; 95% CI, 0.75 to 1) with CD (*n* =34), whereas the AUC was 0.89 (sensitivity of 0.88 and specificity of 0.90; 95% CI, 0.74 to 1) with UC (*n* =31) (Fig. 4B). Splitting the IBD group decreased the sensitivity but increased the specificity from 0.66 to 0.88 for CD and 0.90 for UC.

To discriminate CD (*n* = 34) from UC (*n* = 31), we built a model based on predictor variables collected at baseline for both groups that included fungal and bacterial loads, serum hemoglobin, fecal calprotectin levels, and number of previous relapses. The AUC on the representative test set was 0.76 with a sensitivity of 0.82 and a low specificity of 0.7 (95% CI, 0.56 to 0.95) (Fig. 4C). To discriminate UC (*n* = 31) from CD (*n* = 34), variables such as fungal and bacterial loads, previous number of relapses, and CRP and calprotectin levels led to the highest AUC of 0.86, sensitivity of 0.9, and specificity of 0.82 (95% CI, 0.7 to 1) (Fig. 4C).

To predict flare in CD patients (*n* = 34), we used the data sets collected at baseline and the disease severity status after 1 year of follow up: 21 CD patients remained in remission and 13 relapsed. The most important predictor variables were bacterial load, CRP and calprotectin levels, and previous number of relapses, which allowed us to achieve the highest AUC of 0.87, sensitivity of 0.75, and specificity of 1 (95% CI, 0.6 to 1) (Fig. 4D). To predict flare in UC (*n* = 31), we also used the data sets collected at baseline and the disease severity status after 1 year of follow up, during which 13 UC patients remained in remission and 18 relapsed. The most important predictors were bacterial load, fungal-to-bacterial-load ratio, weight, gender, previous number of relapses, and smoking habit, for which we achieved an AUC of 0.83, sensitivity of 0.67, and specificity of 1 (95% CI, 0.51 to 1) (Fig. 4D).

We finally evaluated the impact of standard markers such as calprotectin and CRP, measured only in IBD patients, in the predictions of relapse. For this purpose, we built two models with and without the markers. As shown in Fig. S1 in the supplemental material, predictions decreased when CRP and calprotectin were removed from the model (AUC decreased from 0.87 to 0.62 for CD and from 0.8 to 0.73 for UC).

10.1128/mSystems.01277-20.1 FIG S1 Receiver operating characteristic (ROC) curves for the models testing calprotectin and CRP. (A) ROC curve for CD. (B) ROC curve for UC. The models either combined CRP and calprotectin with other markers such as bacterial and fungal loads (Combination, green line) or did not take into account calprotectin and CRP (Without CRP and Calpro, blue line). Download FIG S1, PDF file, 0.05 MB. Copyright © 2021 Sarrabayrouse et al. 2021 Sarrabayrouse et al. https://creativecommons.org/licenses/by/4.0/ This content is distributed under the terms of the Creative Commons Attribution 4.0 International license.

From all the models constructed, the analyses indicated that combining microbial data with other laboratory and demographic data improved the predictions by 18% (from an average AUC of 0.842 [95% CI, 0.65 to 0.98]).

## DISCUSSION

Microbiome sequence data have been proposed to distinguish IBD from non-IBD, and CD from UC, and to predict disease relapse and led to microbiomarkers for diagnosis and prognosis (5, 16, 17). Other studies used qPCR to quantify specific groups of bacteria to characterize these differences (13). We recently proposed mucosal bacterial load as a biomarker to stratify CD patients for fecal microbiota transplantation (18). The present study applied qPCR to estimate the number of fungal and bacterial cells in stool samples of CD and UC patients and healthy controls who were related and nonrelated to the IBD patients and to use the loads as predictive biomarkers.

First, independently of health status, we estimated that the number of fungal cells accounts for 0.04% of the bacterial cells, which is very close to the 0.03% of the fecal microbiota previously proposed by Qin et al. (2) To the best of our knowledge, our study is the first to provide an estimation of fungal cells on the basis of the copy number of the ITS2 gene.

Our findings complement previous reports indicating that relatives of IBD patients present an altered gut microbial community compared to that of nonrelated healthy controls. Indeed, Ijaz et al. showed that the gut microbiome composition and functions of healthy relatives of CD patients were altered but to a lesser extent than the members of their families with this condition (19). Moreover, the work by Varela et al. indicated that the unaffected relatives of UC patients carry a lower level of Faecalibacterium prausnitzii than nonrelated healthy controls (14).

Based on microbiome sequence data, CD and UC patients have been shown to display compositional and functional alteration of the gut microbiome (5, 16, 20). Also, at the taxonomic level, ileocolonic CD patients present a greater dysbiosis than individuals with colonic CD (16) and a greater variability over time than UC cases (5) In this study, we complemented these observations, revealing that CD patients differ from UC patients and from the unaffected relatives of UC. Interestingly, unaffected relatives of CD did not show significant differences in both fungal and bacterial loads from their affected family members, and UC patients showed differences from their relatives only in bacterial load. These observations validate previous findings that the environment or/and genetic traits associated with dysbiosis play a role in IBD pathogenesis (21). Another difference between CD and UC patients was the variability of fungal and bacterial loads as the disease progressed. This instability was revealed by the increase in both fungal and bacterial loads when CD patients relapsed, whereas UC patients did not present differences between remission and relapse states. This particularity in CD patients complements our previous findings reported in the work of Pascal et al. (5), in which these patients showed greater UniFrac indexes, thereby indicating higher taxonomic variability than UC patients over time and when individuals relapsed.

On the basis of higher UniFrac distances of sequence data obtained from fecal samples, our previous study showed that CD patients were more dysbiotic than UC ones compared to healthy controls. These sequence data led to the design of microbial signatures to discriminate CD from non-CD based on the relative abundance of eight bacterial genera (5). However, the similarity of microbiome composition between UC patients and healthy controls prevented the identification of microbiomarkers. Using microbial loads, the present study provides another perspective on the use of fecal microbiota to differentiate CD from non-CD and UC from non-UC. Our findings, in particular with CD, were validated by another patient cohort recruited in Belgium, thereby discarding geographical differences between two European countries in terms of fungal and bacterial loads.

Laboratory biomarkers, in particular fecal calprotectin, have been proposed as an inexpensive, safe, and reliable test in monitoring disease activity and response to therapy as well as in predicting relapse, postoperative recurrence, or pouchitis (22, 23). However, the laboratory biomarkers do not differentiate CD from UC or IBD from non-IBD, and concerns were raised regarding the choice of the optimal cutoff level to make it universal (24). We demonstrated in this study that fecal calprotectin and CRP could be useful when combined with microbial loads not only to improve relapse prediction for both CD and UC but also to discriminate CD from UC.

This study has several limitations. First, we used CD patients mainly affected in the ileum or ileocolonic region of the GI tract, a feature that might not be generalized to all CD subtypes, such as those affecting mainly the colon. Second, we recruited IBD patients who were not newly diagnosed adult patients. In this regard, their microbial community may have been shaped over time by the overreaction of their immune systems and by treatments. Third, we used the ITS2 system, one of the commonly used techniques to analyze fungal load, and not the other most used technique, which is the 18S rRNA system. Indeed, there is still a debate on which method is more appropriate for fungal analysis. However, a kind of consensus has been reached for the preferred used of the ITS region instead of the 18S rRNA gene due to the amplification of nonfungal species including DNA from food and from the host by primers targeting the latter system (25). Fourth, random forest models were applied to relatively small groups of subjects. A future validation study may recruit other subtypes of CD, newly diagnosed and pediatric subjects, and also non-IBD chronic intestinal disorders and extend the cohort size. Apart from being noninvasive, one of the main advantages of the use of microbial load as a biomarker is that fecal sample collection and qPCR are techniques that are relatively easy to implement in a clinical setting.

Finally, here we demonstrated the capacity of a machine learning technique, namely, random forest, to classify unaffected relatives of IBD patients, discriminate CD from UC, and predict disease relapse with a good average performance. Therefore, when further validated by a more extended population-based cohort study, we believe that fungal and bacterial loads, combined with other standard laboratory tests (CRP and fecal calprotectin) and demographic data, could provide physicians with a noninvasive and highly efficient test through which to discriminate disease subtypes or predict disease flare in the hospital setting.

## MATERIALS AND METHODS

### Study population.

We used fecal samples collected in two previous Spanish and Belgian IBD retrospective cohorts. The characteristics of the IBD patients are described in the work of Pascal et al. (5) and Machiels et al. (15) (see Table S1 in the supplemental material). A total of 294 fecal samples were obtained from 206 participants. The Spanish cohort consisted of 28 nonrelated healthy controls (NRHC), 29 healthy control relatives of CD patients (HCR[CD]), 29 healthy control relatives of UC patients (HCR[UC]), and 34 CD and 31 UC patients recruited in the University Hospital Vall d’Hebron in Barcelona, Spain (MetaHIT).

For the Spanish cohort, inclusion criteria included a diagnosis of UC and ileal or ileocolonic CD confirmed by previous endoscopy and histology. Clinical remission for at least 3 months was diagnosed based on the validated colitis activity index (CAI) for UC and the CD activity index (CDAI) for CD, stable maintenance therapy (either aminosalicylates, azathioprine, or no drug), and a history of at least three clinical recurrences in the past 5 years. All healthy controls had no history of chronic disease. At inclusion and during the follow-up, we collected the following data: diagnostic criteria; location and behavior of CD; extension of UC; and standard laboratory and demographic data, including blood cell count, CRP, hemoglobin levels, fecal calprotectin, smoking habit, gender, height, and weight. Clinical recurrence was defined by a value of 4 or higher for the CAI and higher than 150 for the CDAI. Exclusion criteria included pregnancy or breast-feeding; severe concomitant disease involving the liver, heart, lungs, or kidneys; and treatment with antibiotics during the previous 4 weeks.

For the Belgian cohort, 55 patients with active CD, who underwent an ileocecal resection, were prospectively recruited between 2011 and 2016 via the University Hospitals Leuven (Belgium) (Table S1). Fecal samples were provided before surgery.

### Ethical considerations.

For both the Spanish and Belgian cohorts, the protocols were submitted and approved by the local Ethical Committee of the University Hospital Vall d’Hebron (Barcelona, Spain) and of the University Hospital Gasthuisberg (Leuven, Belgium), respectively. All volunteers received information concerning their participation in the study and gave written informed consent.

### Demographic and standard laboratory data used as predictor variables.

Demographic data included gender, age, height and weight, body mass index (BMI), smoking history, age at disease onset, and number of previous relapses. Standard laboratory data included blood count, serum CRP and hemoglobin levels, fecal fungal and bacterial loads, and fungal-to-bacterial load ratio. Fecal calprotectin was measured as previously described in the work of Pascal et al. (5).

### Microbial load analysis. (i) Fecal sample collection and genomic DNA extraction.

Fecal samples collected in Spain and Belgium in the previous studies were immediately frozen by the participants in their home freezer at −20°C and later brought to the laboratory in a freezer pack, where they were stored at −80°C. Genomic DNA was extracted following the recommendations of the International Human Microbiome Standards (IHMS) (26). A frozen aliquot (250 mg) of each sample was suspended in 250 μl of guanidine thiocyanate, 40 μl of 10% *N*-lauroyl sarcosine, and 500 μl of 5% *N*-lauroyl sarcosine. Genomic DNA was extracted by mechanical disruption of the microbial cells with beads, and nucleic acids were recovered from clear lysates by alcohol precipitation.

### (ii) Generating a recombinant ITS2 plasmid to create a standard curve for quantification by real-time PCR (qPCR).

The internal transcribed spacer 2 (ITS2) region of fungi was amplified by PCR using the genomic DNA extracted as described above. Specific primers targeting the ITS2 gene were used for amplification: ITS2-fungi-sense (5′-GTG ART CAT CGA ATC TTT-3′) and ITS2-fungi-antisense (5′-GAT ATG CTT AAG TTC AGC GGG T-3′) (8). PCR was carried out following the procedure below: denaturation at 95°C for 2 min, followed by 35 amplification cycles at 95°C for 30 s, at 55°C for 30 s, and at 72°C for 60 s, and a final extension cycle at 72°C for 10 min. Serial dilutions of DNA, ranging from 10^−1^ to 10^−4^, were tested in an attempt to identify the appropriate concentration of the template that would optimize the PCR amplification system, reaching a balance between the smallest amount of potential PCR inhibitors and the highest detectable amount of DNA template. The PCR product obtained from the 10^−1^ DNA dilution template provided the optimal PCR amplification and was then used for ligation into the pCR 2.1-TOPO cloning vector (Thermo Fisher Scientific, Madrid, Spain) and for bacterial transformation in the Top10F strain of Escherichia coli. After culturing the transformed bacteria, we randomly picked up 20 colonies that we recultivated in LB liquid medium to perform plasmid extraction. We randomly selected five extracted plasmids and performed the ITS2 PCR as previously described. PCR results showed three ITS2 regions of different sizes inserted into the five plasmids. We tested each of them for their ability to provide a real-time PCR standard curve. Serial dilutions of the ITS2 plasmid, with concentrations ranging from 10^6^ to 10° copies/reaction, were prepared, and the fungal ITS2 region was amplified using ITS2-fungi-sense (5′-GTG ART CAT CGA ATC TTT-3′) and ITS2-fungi-antisense (5′-GAT ATG CTT AAG TTC AGC GGG T-3′) primers. The real-time PCR amplifications were done using the ITS2 PCR program, under the following conditions: denaturation at 95°C for 2 min, followed by 40 amplification cycles at 95°C for 30 s, at 55°C for 30 s, and at 72°C for 60 s, and a final extension cycle at 72°C for 10 min. Real‐time PCR software (Applied Biosystems, Foster City, CA, USA) was used to calculate the slope and the *R*^2^ (Pearson coefficient of determination) to evaluate the standard curve efficiency of each plasmid. A satisfactory reaction should have an efficacy of between 90% and 110%, which corresponds to a slope of −3.58 to −3.10 and an *R*^2^ value between 1 and 0.90. For each plasmid, we repeated the standard curve assay three times and finally selected the plasmid HD8, which showed the greatest efficiency over the three experiments. We confirmed the reproducibility of the efficiency of the standard curve obtained with HD8 by repeating the standard curve assay several times.

### (iii) ITS2 and 16S rRNA PCR amplification.

Fungal and bacterial loads were estimated in fecal samples by quantifying the ITS2 region and 16S rRNA gene by qPCR, respectively. Amplification was carried out using a 7500 Fast real‐time PCR system (Applied Biosystems, Foster City, CA, USA). The fungal ITS2 region was amplified using ITS2-fungi-sense (5′-GTG ART CAT CGA ATC TTT-3′) and ITS2-fungi-antisense (5′-GAT ATG CTT AAG TTC AGC GGG T-3′) primers. The V4 hypervariable region of the 16S rRNA genes (290 bp) was amplified using the following primers: V4F_517_17 (5′‐GCC AGC AGC CGC GGT AA‐3′) and V4R_ 805_19 (5′‐GAC TAC CAG GGT ATC TAA T‐3′) (27). The PCR was performed in a volume of 25 μl using Power SYBR green PCR master mix (Fisher Scientific, Madrid, Spain) containing 100 nM (each) primer. For the 16S rRNA gene, the reaction conditions were 50°C for 2 min, 95°C for 10 min, and 40 cycles of 95°C for 15 s and 60°C for 1 min. For the ITS2 region, the reaction conditions were 95°C for 2 min, followed by 40 cycles at 95°C for 30 s, 55°C for 30 s, and 72°C for 60 s, and a final extension cycle of 72°C for 10 min. All samples were processed in triplicate, and mean values were calculated. Melting curves were performed and inspected after amplification to evaluate the specificity of the PCR. To generate standard curves, calculated amounts of linearized plasmids, in which the amplified region from a control bacterium had been inserted, were used. Plasmid concentration was measured using a NanoDrop ND‐1000 spectrophotometer (Thermo Scientific, Wilmington, DE, USA), and the number of copies was calculated from the molecular weight of the plasmid. Serial dilutions of the template DNA were amplified to extrapolate bacterial (from 10^2^ to 10^7^) and fungal (from 10° to 10^6^) copy numbers. Results were expressed in copies per gram of stool.

### (iv) Statistical analysis and model construction.

To assess differences in the microbial load between groups on the basis of disease status or disease evolution, we used the nonparametric Wilcoxon signed-rank test (pairwise paired comparisons) and Mann-Whitney U test (pairwise unpaired comparisons). We considered a type I error of 5%. To evaluate the relation between clinical and/or experimental variables, we calculated nonparametric Spearman correlation index.

For different disease prediction models such IBD diagnosis, CD and UC discrimination, and CD and UC relapse prediction, we used random forest, a supervised learning algorithm that randomly constructs and merges multiple decision trees on different bootstrap samples into one “forest” at the training step. To build each random forest model, we used the number of trees that provided the highest AUC (area under the receiver operating characteristic [ROC] curve). The following predictor variables were used in models that included IBD and non-IBD cohorts: age, BMI, gender, smoking status, fungal and bacterial loads, and fungal-to-bacterial load ratio. The variables of models that involved only IBD patients were as follows: age; BMI; gender; smoking habit; microbial data; height; weight; age at disease onset; number of previous relapses; smoking history; CRP, hemoglobin, and calprotectin levels; and white blood cell count.

### Data availability statement.

All data are incorporated into the article and its supplemental material.
