# Supplementary material for: Fungal and Bacterial Loads: Noninvasive Inflammatory Bowel Disease Biomarkers for the Clinical Setting
Source: mSystems. 2021 Mar 23;6(2):e01277-20. doi: 10.1128/mSystems.01277-20 (PMC8547002; doi:10.1128/mSystems.01277-20)
Supplement: TABLE S1 [file msystems.01277-20-st001.pdf]

Table S1. Characteristics of participants

Baseline clinical characteristics	CD Spanish cohort (n=34)	CD Healthy Relatives Spanish cohort (n=29)
Male/female (%)	13/21 (38.2/61.7)	12/17 (41.3/58.7)
Median (IQR) age at surgery (years) or at sample collection	34 (18 – 58)	36 (18 – 58)
Median duration of disease (IQR) at surgery (years) or at sampling	6.5 (0 – 28)	
Maximum disease location (Montreal classification)		
L1 ileal (%)	12 (35)	
L2 colonic (%)	0 (0)	
L3 ileocolonic (%)	22 (64.7)	
L4 isolated upper disease (%)	2 (5.8)	
Disease behaviour at surgery (Montreal classification)		
B1 non-stricturing, non-penetrating (%)	3 (8.8)	
B2 stricturing (%)	22 (64.7)	
B3 penetrating (%)	5 (14.7)	
p perianal disease (%)	3 (8.8)	
Active smoking at surgery (%)	10 (29.4)	
Medication at surgery or at sampling		
Mesalamine–sulfasalazine (%)	4 (11.8)	
Corticosteroids (%)	2 (2.9)	
Immunosuppressants (%)	14 (41.1)	
Anti-TNF (%)	12 (23.5)	
Antibiotics (%)	0 (0)	
Methotrexate	1 (2.9)	
Other	10 (29.4)	
None	1 (2.9)	

Baseline clinical characteristics	CD Belgian cohort (n=55)
Male/female (%)	28/25 (52.8/47.2)
Median (IQR) age at surgery (years) or at sample collection	41.3 (26.5 – 52.9)
Median duration of disease (IQR) at surgery (years) or at sampling	15.7 (4.1 – 27.1)
Maximum disease location (Montreal classification)	
L1 ileal (%)	18 (34)
L2 colonic (%)	0 (0)
L3 ileocolonic (%)	35 (66)
L4 isolated upper disease (%)	2 (3.8)
Disease behaviour at surgery (Montreal classification)	
B1 non-stricturing, non-penetrating (%)	2 (3.8)
B2 stricturing (%)	21 (39.6)
B3 penetrating (%)	30 (56.6)
p perianal disease (%)	15 (28.3)
Active smoking at surgery (%)	16 (30.2)
Medication at surgery or at sampling	
Mesalamine–sulfasalazine (%)	4 (7.5)
Corticosteroids (%)	10 (18.9)
Immunosuppressants (%)	12 (22.6)
Anti-TNF (%)	7 (13.2)
Antibiotics (%)	9 (16.9)
Methotrexate	
Other	
None	

Baseline clinical characteristics	UC Spanish cohort (n=31)	UC Healthy Relatives Spanish cohort (n=29)
Male/female (%)	9/24 (27.2/72.7)	9/20 (31.03/68.96)
Median (IQR) age at sample collection	43 (24 – 62)	38 (22 – 60)
Median duration of disease (IQR) at sampling	9 (1 – 23)	
Disease behaviour at sampling		
E1 proctitis	9 (27.3)	
E2 left sided colitis	11 (33.3)	
E3 pancolitis	11 (33.3)	
Medication at sampling		
Mesalamine (%)	11 (24)	
Corticosteroids (%)	2 (6)	
Immunosuppressants (%)	8 (24)	
Other	2 (6)	
None		

Characteristics	Related Helathy Controls (n=28)
Age (years)	27 (20 - 64)
Weight (Kg)	59,75 (48,5 - 92)
Height (cm)	168 (154 - 192)
BMI(kg/m2)	21.4 (19.2 - 28.6)
**Sex**	
Female	19 (68%)
Male	9 (32%)
**Smoker**	
Non-smoker	21 (75%)
Smoker	1 (3.57%)
Unknown	6 (21.42%)
