# Supplementary figures and images for: Fungal and Bacterial Loads: Noninvasive Inflammatory Bowel Disease Biomarkers for the Clinical Setting
Source: mSystems. 2021 Mar 23;6(2):e01277-20. doi: 10.1128/mSystems.01277-20 (PMC8547002; doi:10.1128/mSystems.01277-20)

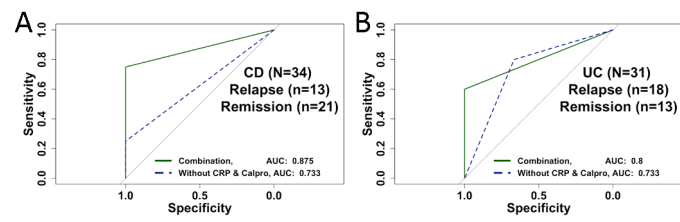

Supplement: FIG S1 [file msystems.01277-20-sf001.pdf]
